# The Relationship between Motor Skills, Perceived Social Support, and Internalizing Problems in a Community Adolescent Sample

**DOI:** 10.3389/fpsyg.2016.00543

**Published:** 2016-04-22

**Authors:** Vincent O. Mancini, Daniela Rigoli, Brody Heritage, Lynne D. Roberts, Jan P. Piek

**Affiliations:** ^1^School of Psychology and Speech Pathology, Curtin UniversityPerth, WA, Australia; ^2^School of Psychology and Exercise Science, Murdoch UniversityPerth, WA, Australia

**Keywords:** motor skills, adolescents, motor development, internalizing problems, anxiety, depression, movement

## Abstract

**Objectives:** Poor motor skills are associated with a range of psychosocial consequences, including internalizing (anxious and depressive) symptoms. The Elaborated Environmental Stress Hypothesis provides a causal framework to explain this association. The framework posits that motor skills impact internalizing problems through an indirect effect via perceived social support. However, empirical evaluation is required. We examined whether motor skills had an indirect effect on anxious and depressive symptoms via perceived family support domains.

**Methods:** This study used a community sample of 93 adolescents (12–16 years). Participants completed measures of motor skills, perceived social support across three dimensions (family, friend, and significant other), depressive symptoms, and anxious symptoms. Age, gender, verbal IQ, and ADHD symptoms were included as control variables.

**Results:** Regression analysis using PROCESS revealed that motor skills had an indirect effect on depressive symptoms via perceived family support, but not by perceived friend support or significant other support. The negative association between motor skills and anxious symptoms was not mediated by any perceived social support domain.

**Conclusions:** Findings are consistent with previous literature indicating an association between motor skills and internalizing problems. However, we identified a different pattern of relationships across anxious and depressive symptoms. While anxiety and depressive symptoms were highly correlated, motor skills had an indirect effect on depressive symptoms via perceived family support only. Our findings highlight the importance of family support as a potential protective factor in the onset of depressive symptoms. This study provides partial support for the Elaborated Environmental Stress Hypothesis, however further research is required.

## Background

Motor skills have an important connection with psychosocial wellbeing. Studies have demonstrated that children who have clinical motor impairments, such as developmental coordination disorder (DCD), are at greater risk of experiencing poor psychosocial outcomes, compared to their non-DCD peers. This includes less enjoyment in daily tasks (Bart et al., 2011), low self-esteem (Miyahara and Piek, 2006), less developed social support and friendships (Smyth and Anderson, 2000; Skinner and Piek, 2001), poor social skills (Kanioglou et al., 2005), social isolation and social problems (Smyth and Anderson, 2000; Chen et al., 2009), academic underachievement (Alloway, 2007), peer victimization/bullying (Campbell et al., 2012), decreased quality of life (Hill et al., 2011), withdrawal (Chen et al., 2009), physical inactivity, and obesity (Cairney et al., 2005). In addition, poor motor skills are also associated with symptoms of internalizing disorders such as depression and anxiety (Skinner and Piek, 2001; Francis and Piek, 2003; Lingam et al., 2012; Cairney et al., 2013). This association between poor motor skills and internalizing symptoms could be attributed to common neurodevelopmental etiology between motor skills and emotional regulation (Nicolson et al., 2001; Ekornas et al., 2010). However, each of the previously identified psychosocial consequences of poor motor skills are well-established risk factors for the development of internalizing problems in their own right. Cairney et al. (2013) posited that poor motor skills give rise to these various psychosocial consequences, which in turn results in increased internalizing problems, and these relationships form the basis of their recently proposed Elaborated Environmental Stress Hypothesis. This theoretical framework proposes that the relationship between motor skills and internalizing symptoms is predominantly indirect; poor motor skills may lead to various psychosocial consequences in the individual's surrounding environment (e.g., lower social support, low self-competence, peer victimization, etc.) which subsequently gives rise to increased internalizing symptoms (see Cairney et al., 2013). The model places emphasis on environmental, rather than biological, factors linking motor skills to internalizing problems. In a monozygotic twin study by Piek et al. (2007), twins with DCD were compared to their unaffected co-twin, granting the ability to control for shared genetic factors as well as shared environment. Results demonstrated higher levels of depressive symptoms in the DCD twin compared to their unaffected co-twin. These findings provide support for the Elaborated Environmental Stress Hypothesis; the authors concluded that the differences in depressive symptoms are due to unique environmental stressors associated with DCD.

The Elaborated Environmental Stress Hypothesis (see Cairney et al., 2013, p. 233 for a visual representation) is comprised of multiple direct, mediating, and moderating pathways between motor skills and internalizing symptoms. The complexity of this model has resulted in studies evaluating parts of the model, rather than the model in its entirety. These recent studies have provided support for various pathways embedded within the broader Elaborated Environmental Stress Hypothesis. For example, Wilson et al. (2013) found that the relationship between motor skills and internalizing symptoms in a community sample of young children (4–6 years of age) was mediated by social skills. The authors identified that higher levels of motor skills were associated with higher levels of social skills, which in turn related to a decrease in internalizing symptoms. Rigoli et al. (2012) found the relationship between motor skills and internalizing problems was mediated by levels of self-concept in a normative adolescent sample, another key pathway embedded within the broader Elaborated Environmental Stress Hypothesis. The results of a recent randomized control trial (RCT) by Piek et al. (2015) provided the first intervention study to empirically evaluate the Elaborated Environmental Stress Hypothesis. The authors evaluated the efficacy of the *Animal Fun* universal intervention program aimed at promoting motor and social development in young children (aged 4–6 years). Results demonstrated a lasting increase in prosocial behavior in the intervention group, but not the control group; this increase remained at 6-month and 18-month follow up. The relationships between observed variables were consistent with the pattern of relationships posited by the Elaborated Environmental Stress Hypothesis. These recent empirical developments provide preliminary support for the various pathways specified in the broader causal framework. However, given the recent conceptualization of the model, not all of the pathways contained in the model have yet been empirically evaluated. Therefore, it is important to continue to provide ongoing evaluation of the model.

Early research in the area of poor motor skills often used clinical samples of children with DCD to identify the associated psychosocial consequences (Skinner and Piek, 2001). While studies employing this comparative approach continue to provide important findings, motor skills are best understood as a continuous, rather than dichotomous construct (Wassenberg et al., 2005). More recent developments in the literature have highlighted that the psychosocial implications of motor skills are not limited to children with DCD, but are present across the broader continuum of motor skills (Rigoli et al., 2012; Wilson et al., 2013; Piek et al., 2015). There is a negative linear relationship between internalizing symptoms and motor skills, across the full continuum of movement. In other words, poorer motor skills are associated with increased internalizing symptoms and better movement ability is associated with lower internalizing symptoms. Studies that have utilized broader community samples are able to address the methodological problems that exist when attempting to dichotomize motor skills into clinical (DCD) and non-clinical (not DCD) categories (Hattori et al., 2006). Furthermore, focussing on the extreme end of the motor skill continuum may overestimate the relationship between constructs in the wider population (Rigoli et al., 2012).

Motor skills are considered to be a relatively stable construct, with a predictable pattern of development, stability, and decline over the lifespan (Leversen et al., 2012). The majority of motor coordination literature focuses on motor development in childhood. Childhood represents a critical period for motor development, and often the period in which atypical motor skills are noticed. However, poor motor skills in childhood can persist into adolescence and later life; therefore it is important to consider older populations. Recent literature has identified that the psychosocial implications of poor motor skills extend beyond childhood, into adolescence (Skinner and Piek, 2001; Sigurdsson et al., 2002; Rigoli et al., 2012; Viholainen et al., 2014) and adulthood (Hill and Brown, 2013; Poole et al., 2015). Longitudinal studies have also identified that childhood motor problems can predict psychosocial problems (including internalizing disorders) in later life (Sigurdsson et al., 2002; Lingam et al., 2012; Poole et al., 2015). Skinner and Piek (2001) suggested that the psychosocial consequences of poor motor skills may become more pronounced with age; adolescents with DCD reported higher levels of anxious symptoms than children with DCD. The adolescent DCD group also reported higher levels of anxiety compared to the adolescents without DCD. A secondary finding was that participants with DCD reported lower levels of perceived social support when compared to their non-DCD peers. A limitation of this study was that participants were dichotomized into DCD and non-DCD groups, therefore a linear relationship between motor skills, perceived social support, and internalizing problems could not be tested.

The role of social support in the etiology of internalizing problems in adolescence is well-established. High levels of perceived social support may also act as a protective factor in the onset of internalizing symptoms. Inversely, lower levels of perceived social support are associated with increased internalizing symptoms. In their community sample of 390 students aged 10–15 years, Stewart and Suldo (2011) identified perceived social support from parents, peers, and teachers each uniquely predicted variance in internalizing symptoms. However, perceived parent support emerged as the strongest predictor of all indicators, accounting for 6% of unique variance in their regression model. Similarly, the authors also identified parental support to be the largest unique predictor of externalizing behavior, and also life satisfaction. Rueger et al. (2010) also investigated the impact of perceived social support from various sources (parents, teachers, classmates, close friends, and school) on levels of anxiety and depression in middle school students. Perceived parental support was the most important predictor of all outcomes. As individuals receive support from multiple sources, it is important to consider perceived social support as a multi-factorial construct (Uchino et al., 1996). This approach can also help to identify the most important types of perceived social support.

Anxious and depressive symptoms are often considered concomitantly within the literature. This reflects their shared etiology, similar presentation of symptoms, and high levels of comorbidity (Brady and Kendall, 1992; Kessler and Walters, 1998; Seligman and Ollendick, 1998). The Elaborated Environmental Stress Hypothesis as described by Cairney et al. (2013) conceptualizes internalizing problems as an umbrella term comprised of both anxious and depressive symptoms. Consequently, some studies in the motor coordination literature measure internalizing problems as a single variable (Wilson et al., 2013), or as a latent factor driven by two observed constructs: anxious symptoms and depressive symptoms (Rigoli et al., 2012). However, within the social support literature, studies have identified different relationships between perceived social support and anxious and depressive symptoms. Perceived social support is often found to be associated with depressive symptoms, but less consistently associated with anxious symptoms (Haeffel and Mathew, 2010; Rueger et al., 2010; Väänänen et al., 2014). Consequently, research investigating the relationship between perceived social support and internalizing symptoms should consider anxious and depressive symptoms as separate outcome variables.

Through the reviewed evidence, a relationship between motor skills, perceived social support, and both anxious and depressive symptoms in adolescence has been identified. The Elaborated Environmental Stress Hypothesis provides a model in which these relationships may be framed. It posits perceived social support as a key intermediary factor between motor skills and internalizing symptoms. Lower levels of motor skills are associated with lower perceived social support, which then gives rise to increased levels of internalizing symptoms. In other words, this particular section of the Elaborated Environmental Stress Hypothesis suggests that motor skills has an indirect effect on internalizing problems via perceived social support. However, this is yet to be empirically tested.

## Study aims

The aim of the present study was to empirically evaluate a portion of the Elaborated Environmental Stress Hypothesis by Cairney et al. (2013). Specifically, we tested the proposed indirect pathway from motor skills to internalizing symptoms via perceived social support, in a community sample of adolescents. We also controlled for potential confounding variables of attention deficit/hyperactivity disorder (ADHD), verbal IQ (VIQ), age and gender. ADHD has demonstrated similar psychosocial consequences as DCD (Piek et al., 2007) and has high rates of comorbidity with DCD (Goulardins et al., 2015). We included a parent-report measure that allowed us to statistically account for ADHD symptoms. Verbal IQ (VIQ) was first included as a screening tool to exclude participants with general delayed development (Lingam et al., 2012; Rigoli et al., 2012). Excluding these participants is also important for the present study which utilizes self-report measures. Previous literature has also identified a negative association between VIQ and internalizing symptoms (Rajput et al., 2011). Therefore, we also included VIQ as a control variable. Age and gender were also included as control variables based on previous findings (Sigurdsson et al., 2002; Lingam et al., 2010, 2012).

Based on the reviewed literature, we propose two hypotheses. Hypothesis one states that after controlling for age, gender, VIQ, and ADHD symptoms, motor skills will have an indirect effect on depressive symptoms, via perceived social support from friends, family, and a significant other. Hypothesis two states that after controlling for these same control variables, motor skills will have an indirect effect on anxious symptoms via each of the three domains of perceived social support.

## Methods

### Participants

A community sample of 93 adolescents aged 12–16 years (*M* = 14.21 years, *SD* = 1.09) took part in the present study. Participants completed a battery of cognitive, psychosocial, and motor skills assessments as part of a larger research project (Rigoli et al., 2012). There were 55 males and 38 females. Participants were recruited through a combination of public advertisements and through 5 randomly selected secondary schools located in metropolitan Perth, Western Australia. Participants were required to have a Verbal Comprehension Index (VCI) score of 70 or above on the Wechsler Intelligence Scale for Children-IV (WISC-IV; Wechsler, 2003). This was to exclude any adolescents whose difficulties may be attributed to general delayed development (Geuze et al., 2001). All included participants had no diagnosis of physical disability, chronic illness, or medical conditions that impact development.

The a-priori power analysis for a linear multiple regression analysis to test for mediation indicated that a sample size of 109 participants was required in order to detect a medium effect size in a model with 8 predictors (four control variables, motor skills, and the 3 domains of perceived social support). The present sample of 93 participants falls slightly short of this recommended value. However, the robustness of the bootstrapped estimation methods used in the analysis would assist in addressing this potential limitation.

### Measures

#### The movement assessment battery for children second edition (MABC-2)

The MABC-2 (Henderson et al., 2007) is a standardized instrument used to measure and describe movement difficulties in children between 3 and 16 years of age across three age bands (3–6 years; 7–10 years; 11–16 years). Motor coordination is measured across three domains; manual dexterity, aiming and catching ability, and balance, which are combined to provide an indication of overall motor ability. The assessment is independently administered by a trained professional, and takes approximately 30 min to complete the eight tasks. Age-based standardized scores are derived for each of the domains and an overall total test score. A child is deemed to be “at risk” of having a movement difficulty if their total test score places them between the 5th and 15th percentile; scores below the 5th percentile suggest a severe movement difficulty. The present study used the standardized total test score of the MABC-2. Previous validation studies of the MABC-2 have reported the measure to demonstrate good test retest reliability for each domain and total standardized scores, inter-rater reliability, criterion-related and discriminant validity (Henderson et al., 2007).

#### The multidimensional scale of perceived social support (MSPSS)

The MSPSS (Zimet et al., 1988) is a widely used, 12 item self-report measure of perceived social support adequacy. The measure provides a subjective assessment of social support from three subscales: family, friends, and a significant other. Participants are asked to report the extent to which they agree with each statement using a 7-point scale (1 = *very strongly disagree*, to 7 = *very strongly agree*). An example item is “*I get the emotional help and support I need from my family*.” Subscale scores are calculated by averaging all responses, with higher scores indicating a higher degree of perceived social support from that particular source. Each of the three subscales of the MSPSS demonstrates good internal reliability, and the three factor structure has been validated with adolescent populations (Canty-Mitchell and Zimet, 2000; Walker et al., 2002). Cronbach's alpha for the present sample is 0.88 for the family subscale, 0.88 for the friend subscale, and 0.88 for the significant other subscale.

#### The mood and feelings questionnaire—child version (MFQ)

The MFQ (Costello and Angold, 1988) is a 33-item self-report questionnaire designed for children and adolescents to report depressive symptoms experienced over the two weeks prior to completing the questionnaire. Responses are recorded by using a 3-point scale (0 = *not true*, 1 = *sometimes true*, 2 = *true*). Total scores range from 0 to 66 with higher scores indicating higher depressive symptoms. The MFQ was designed for use with both clinical and non-clinical populations of children and adolescents, and has been widely validated (Costello and Angold, 1988; Wood et al., 1995; Kuo et al., 2005). Consistent with previous reports of high internal reliability (Costello and Angold, 1988), Cronbach's alpha for the MFQ in the present study was 0.92, indicating good internal reliability.

#### The spence children's anxiety scale (SCAS)

The SCAS comprises 38 items designed to measure symptoms of anxiety across 6 subscales: panic attack, agoraphobia, separation anxiety, social phobia, physical injury fears, obsessive compulsive disorder, and generalized anxiety. Responses are recorded by using a 4-point scale (0 = *never*, to 3 = *always*). A total SCAS score is calculated by summing the responses to all 38 items. This total score of the child self-report version was used in the present study. The SCAS has been used in samples of adolescents up to 19 years of age and demonstrates good levels of internal reliability (Spence, 1997), test-retest reliability, convergent and discriminant validity (Spence, 1998; Muris et al., 2000; Essau et al., 2002). Cronbach's alpha for the SCAS in the present study was 0.89, indicating good internal reliability.

#### Wechsler intelligence scale for children- fourth edition (WISC-IV)

The WISC-IV (Wechsler, 2003) is a standardized assessment of cognitive ability for children aged 6–16 years 11 months. The WISC-IV provides an indication of cognitive ability across 4 domains: verbal comprehension, perceptual reasoning, working memory, and processing speed. The WISC-IV is widely used, and considered to be the gold-standard in cognitive assessment for children. It has excellent psychometric properties (Wechsler, 2003). We used the VCI subscale of the WISC-IV in the present study.

#### Strengths and weaknesses of ADHD symptoms and normal behavior (SWAN)

The SWAN (Swanson et al., 2006) is a parent-rated assessment of ADHD symptoms. This 18-item measure involves observations based on the last month, asking the parent to rate their child's behavior compared to similarly aged children. A 7-point scale is used, with scores ranging from 3 (*far below average*) to −3 (*far above average*). An overall score is calculated by averaging the total of all 18 items, with higher scores indicating higher ADHD symptoms. The SWAN has been previously supported as an accurate measure of ADHD symptoms in the general population (Martin et al., 2006; Polderman et al., 2007). Cronbach's alpha for the SWAN in the present study was 0.96, indicating excellent internal reliability.

#### Demographic variables

Single item measures of age and gender were also collected.

### Procedure

The study followed the National Health and Medical Research Council of Australia ethical guidelines. Prior to commencing the study, ethics approval was granted by the relevant University Human Research Ethics Committee and the relevant bodies of the participating schools. Informed consent was obtained from both the adolescent participants and their parents. Participants were then independently assessed by a trained assessor over a period of two sessions (approximately 4.5 h in total). The self-report psychosocial questionnaires were completed by participants, and the parent-report questionnaires by parents. Assessments were conducted at either the family home or at university facilities, selected at the discretion of the families.

## Results

Means, standard deviations, ranges, and bivariate correlations for the observed variables in this study are provided in Table 1. Five adolescents (5.4% of the total sample) were identified as having significant movement difficulty on the MABC-2 (at or below the 5th percentile). This is comparable to population estimates of 5–6% (American Psychiatric Association, 2013). Two adolescents were identified as at-risk for movement difficulty (between the 6th and 15th percentile). Ten adolescents scored in the clinical range for depression on the MFQ (a score of 29 and above). Seven participants scored in the subclinical range for anxiety on the SCAS (1 standard deviation above the normative mean), and an additional five participants scored in the clinical range (more than 1.5 standard deviations above the normative mean). Two of the five adolescents identified as having significant movement difficulty scored in the clinical range for both the MFQ and the SCAS. One of the two adolescents identified as at-risk for movement difficulty scored in the subclinical range for the SCAS.

**Table 1:** **Means, standard deviations (*SD*), observed range of scores, and bivariate correlations between variables (*N* = 93)**.

**Variable**	**Descriptives**			**Bivariate correlations (Pearson's** ***r*****)**													
	**Mean**	***SD***	**Range**	**1**	**2**	**3**	**4**	**5**	**6**	**7**	**8**	**9**	**10**	**11**	**12**	**13**	**14**
1. MABC-2 Total Test Score^a^	10.63	2.57	3–16	–													
2. MABC-2 Manual Dexterity^a^	9.57	2.47	3–15	0.657^**^	–												
3. MABC-2 Aiming and Catching^a^	11.03	2.73	4–16	0.656^**^	0.071	–											
4. MABC-2 Balance^a^	11.42	2.98	4–14	0.780^**^	0.264^*^	0.423^**^	–										
5. MSPSS Total^b^^c^	5.72	0.88	2–7	0.242^*^	0.195	0.058	0.302^**^	–									
6. MSPSS Family Support^b^^d^	5.78	1.15	1–7	0.223^*^	0.088	0.180	−0.247^*^	0.753^**^	–								
7. MSPSS Friend Support^b^^d^	5.69	1.05	2–7	0.226^*^	0.223^*^	0.006	0.263^*^	0.758^**^	0.260^*^	–							
8. MSPSS Significant Other Support^b^^d^	5.68	1.11	2–7	0.129	0.160	−0.055	0.213^*^	0.873^**^	0.500^**^	0.585^**^	–						
9. MFQ Total^b^^e^	13.48	10.43	1–48	−0.329^**^	−0.108	−0.319^**^	−0.307^**^	−0.215^*^	−0.347^**^	−0.138	−0.020	–					
10. SCAS Total^b^^e^	21.77	12.49	1–67	−0.324^**^	0.003	−0.371^**^	−0.314^**^	−0.018	−0.106	−0.054	0.119	0.720^**^	–				
11. WISC-IV VCI^a^	106.63	11.25	81–132	0.152	0.075	0.048	0.155	0.055	−0.004	0.117	0.023	−0.155	−0.185	–			
12. Age (Years)	14.21	1.09	12–17	−0.114	−0.069	−0.066	−0.095	−0.054	−0.101	−0.063	0.036	0.161	0.187	−0.167	–		
13. Gender	–	–	–	−0.069	0.235^*^	−0.397^**^	−0.007	0.261^*^	−0.038	0.359^**^	0.319^**^	0.079	0.179	−0.018	0.021	–	
14. ADHD Symptoms^b^^c^	−1.00	1.02	−3.0–1.22	−0.040	−0.143	0.058	0.011	0.051	−0.030	0.120	0.043	0.170	0.029	−0.284^*^	0.093	−0.212	–

*MABC-2, Movement Assessment Battery for Children, Second-Edition; MSPSS, Multidimensional Scale for Perceived Social Support; MFQ, Mood and Feelings Questionnaire; SCAS, Spence Children's Anxiety Scale; WISC-IV, Wechsler Intelligence Scale for Children; VCI, Verbal Comprehension Index; ADHD, Attention Deficity Hyperactivity Disorder*.

* *p < 0.05 (two-tailed)*.

** *p < 0.001 (two-tailed)*.

a *Standard Score*.

b *Raw Score*.

c *Scores are calculated by averaging the total of all items in the measure*.

d *Scores are calculated by averaging the relevant subscale items in the measure*.

e *Scores are calculated by summing all items in the measure*.

### Mediation analysis

Tests of mediation using the PROCESS macro (Hayes, 2013) were conducted in SPSS. The direct and indirect effect of each model were estimated with 10,000 bootstrapped 95% bias-corrected and accelerated (BcA) confidence intervals to assess for statistical significance, as this method is robust to non-normality for the indirect path estimation. Two models were tested, one predicting depressive symptoms and the other predicting anxious symptoms. In each model motor skills scores were specified as the independent variable, and social support from family, friends, and a significant other as the mediator variables, with, gender, age, and ADHD symptoms as covariates.

#### Depressive symptoms

**Figure 1:**
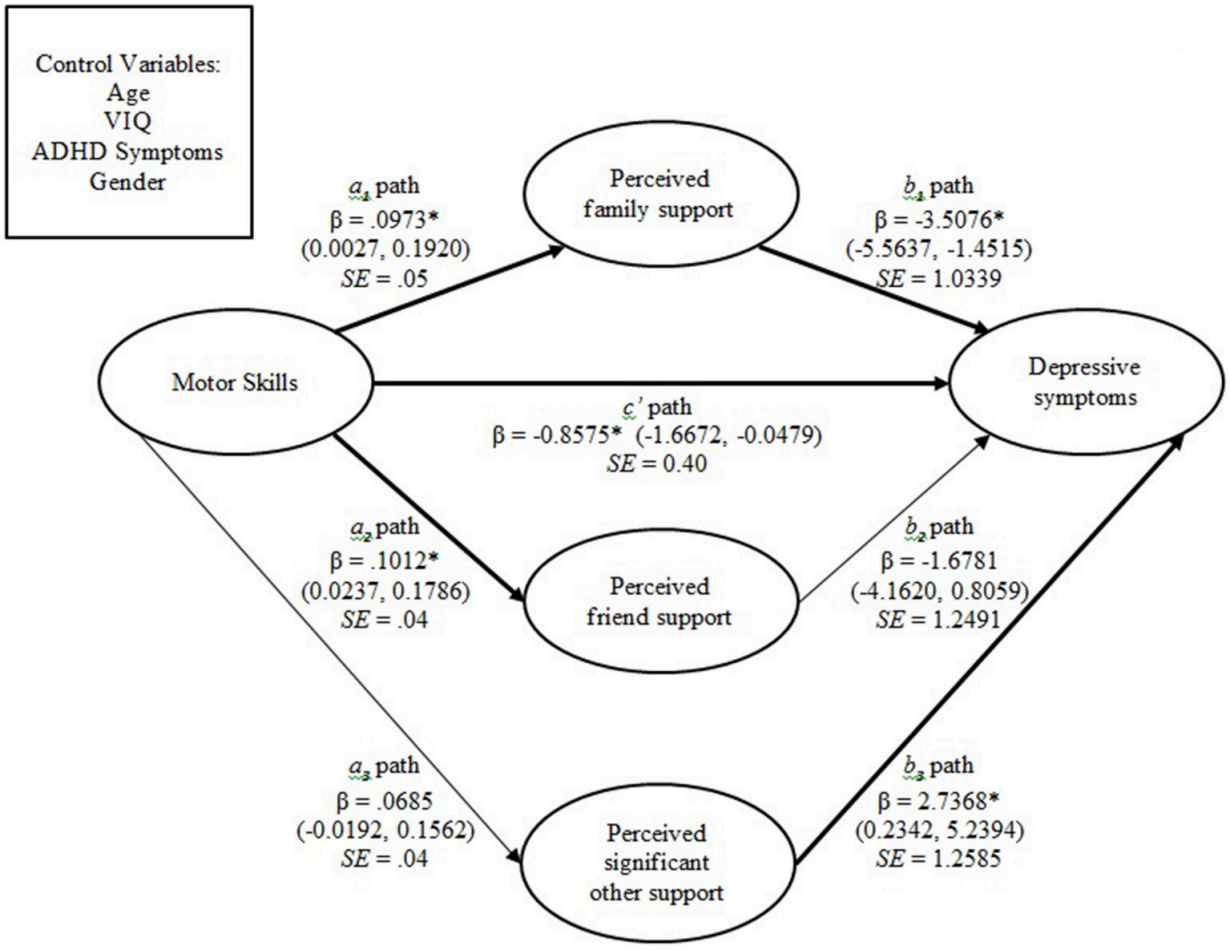
**Mediation model in which perceived family support partially has an indirect effect on the association between motor skills and depressive symptoms**. Significant pathways are depicted in bold. ^*^P < 0.05. Note: 95% bias-corrected confidence intervals provided in parentheses. β, Standardized coefficient; SE, standard error.

In combination, the predictors included in the total model account for approximately 26.36% of the variance in depressive symptoms, Model *R*^2^=0.26 *F*_(8, 84)_ = 3.76, *p* < 0.001, and a large effect (Cohen, 1992). Motor skills did not have an indirect effect on depressive symptoms via perceived friend support, or perceived significant other support, as the confidence intervals of both indirect pathways included zero. There was a significant indirect effect of motor skills on depressive symptoms via perceived family support; *ab* = −0.34, 95% *BcA CI* = −0.90 to −0.024. The association between motor skills and depressive symptoms was lessened but remained significant after the inclusion of the covariates and mediator variables, *c*' = −0.86, 95% *BcA CI* = −1.67 to -0.05. The direct effect, after controlling for the effect of the mediator variables and covariates, was therefore a significant predictor of depressive symptoms. In summary, we identified a direct effect from motor skills to depressive symptoms, and also an indirect effect via perceived family support. These relationships are presented in Figure 1.

#### Anxious symptoms

**Figure 2:**
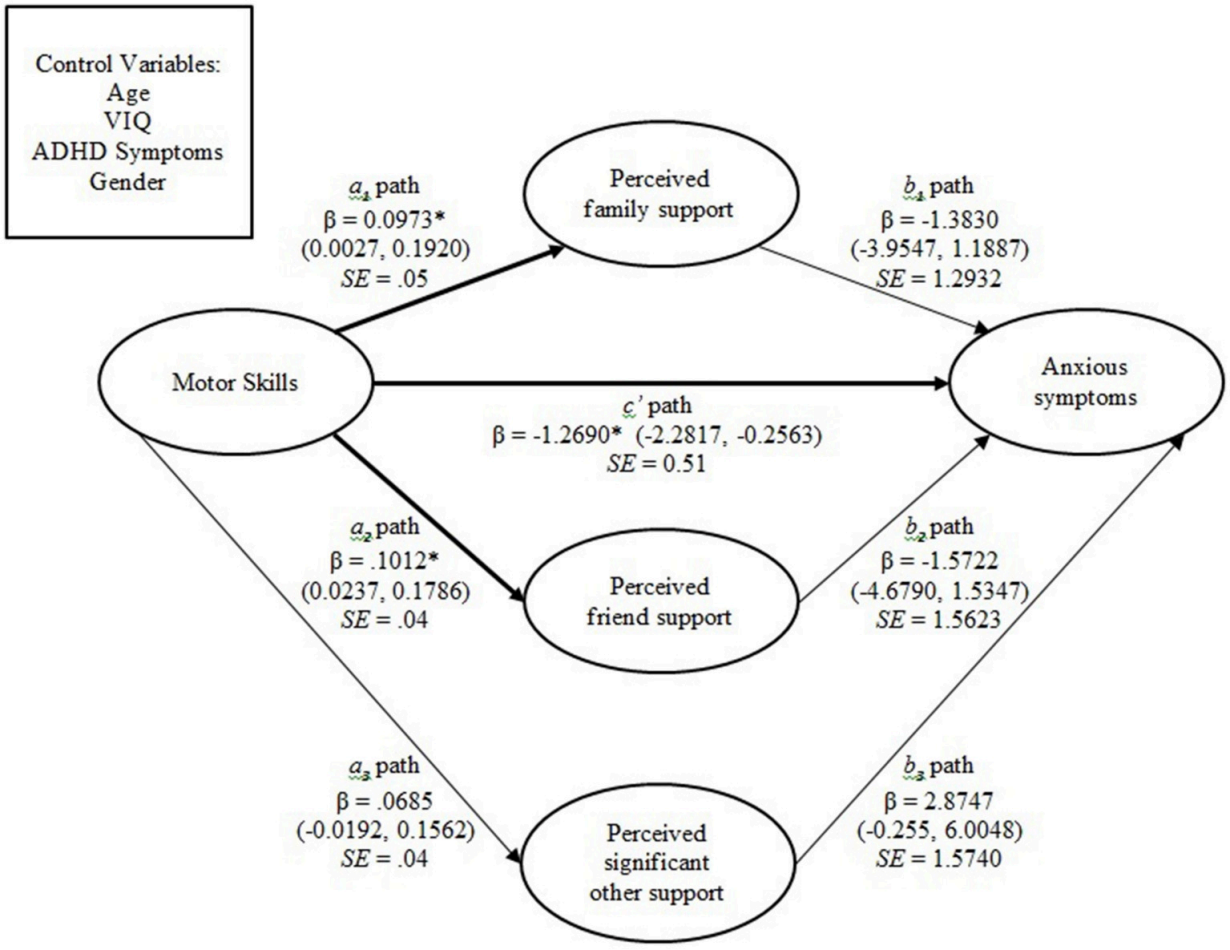
**Mediation model in which there is no indirect relationship between motor skills and anxious symptoms. Significant pathways are depicted in bold**. ^*^P < 0.05. Note: 95% bias-corrected confidence intervals provided in parentheses. β, Standardized coefficient; SE, standard error.

For the mediation model with anxious symptoms as the outcome variable, motor skills, the three domains of perceived social support, and the covariates accounted for approximately 19.77% of the variance in anxious symptoms, Model *R*^2^=0.20, *F*_(8, 84)_ = 2.59, *p* = 0.014, and a moderate to large effect (Cohen, 1992). There was no significant indirect effect of motor skills on anxious symptoms via any domain of perceived social support, as all confidence intervals for the *ab* path estimates included zero within their boundaries. The direct effect of motor skills was significant after accounting for the effect of the mediator variables and the covariates, *c*′ = −1.45, 95% *BcA CI* = −2.44 to −0.46. These relationships are presented in Figure 2.

## Discussion

The aim of the current study was to empirically evaluate a key part of the recently proposed Elaborated Environmental Stress Hypothesis by Cairney et al. (2013). This causal framework posits that poor motor skills give rise to internalizing problems via the intermediary effect of various personal and social factors. Few studies have empirically examined this framework since it was conceptualized. Therefore, the current study is important in adding to existing research. We specifically sought to evaluate whether the relationship between motor skills and internalizing symptoms was mediated by perceived social support in a community adolescent sample. Hypothesis one stated that the relationship between motor skills and depressive symptoms would be mediated by perceived social support from friends, family, and a significant other, after controlling for age, gender, VIQ, and ADHD symptoms. This hypothesis was partially supported. Motor skills had a direct effect on depressive symptoms, and an indirect effect via perceived family support. Hypothesis two stated that the relationship between motor skills and anxious symptoms would be mediated by perceived social support from friends, family, and a significant other, after controlling for age, gender, VIQ, and ADHD symptoms. This hypothesis was not supported. Motor skills had a direct effect on anxious symptoms, but did not have an indirect effect on anxious symptoms via any domain of perceived social support.

Similar studies that have used measures of internalizing symptoms have found comparable results to the present study. For example, Wilson et al. (2013) found social skills mediated the relationship between motor skills and internalizing symptoms in young children. The authors measured internalizing symptoms as a single construct. Other studies have considered measures of anxious and depressive symptoms as observed variables which are driven by the latent construct of internalizing symptoms (Rigoli et al., 2012). The use of internalizing symptoms as an umbrella term for anxious and depressive symptoms is widely supported (Brady and Kendall, 1992; Kessler and Walters, 1998; Seligman and Ollendick, 1998), though there are several distinctions in the etiology and impact of anxious and depressive symptoms. Our findings highlight the need to examine the importance of perceived social support for adolescents on anxious and depressive symptoms separately. Previous studies in adolescent populations have also noted stronger and more consistent negative linear associations between perceived social support and depressive symptoms, compared to anxious symptoms (Haeffel and Mathew, 2010; Rueger et al., 2010; Väänänen et al., 2014). Analyses that unify anxious and depressive symptoms into internalizing symptoms may risk attenuating the relationship between variables. Furthermore, the Elaborated Environmental Stress Hypothesis includes a range of secondary psychosocial consequences that give rise to internalizing problems; it may be important to consider the differential impacts of these other factors on anxious and depressive symptoms.

While both mediation models accounted for significant variance, a substantial proportion of variance in anxious and depressive symptoms was unaccounted for. This suggests that there are additional factors that contribute to the formation of anxious and depressive symptoms in adolescents. We only investigated a portion of the Elaborated Environmental Stress Hypothesis. Multiple factors posited to mediate and/or moderate the association between motor skills and internalizing symptoms were untested in the present study. It is likely that future studies that look at several factors embedded within the Elaborated Environmental Stress Hypothesis will account for additional variance in internalizing symptoms. This is consistent with the understanding that the etiology of internalizing symptoms across the life span is influenced by multiple factors (Koplewicz and Klass, 1993).

Consistent with previous literature, the current study identified a direct association between motor skills and both anxious and depressive symptoms. This relationship is fundamental to the Elaborated Environmental Stress Hypothesis. Cairney et al. (2013) originally developed this framework for use in the child DCD population. However, more recent studies have also provided support for its use in broader community populations (Rigoli et al., 2012; Wilson et al., 2013; Viholainen et al., 2014; Piek et al., 2015). The present study adds to this literature, enlisting a community sample of adolescents and providing partial support for this framework.

In the present study, we specifically focused on perceived social support as a possible secondary consequence. It has been suggested that higher levels of motor skills are associated with higher levels of perceived social support, as it increases the chances for positive peer interactions and formation of friendships (Cairney et al., 2013). Similarly, the presence of poor motor skills may lead to frustration from family members and teachers, who may incorrectly attribute the individual's difficulties as inattention, task avoidance, or laziness (Missiuna et al., 2006). The individual may then perceive a decrease in perceived social support from these sources. Our current findings are congruent with previous research, as we identified a positive linear association between motor skills and perceived social support (from friends and family).

The association between perceived social support and internalizing symptoms in adolescence is well-researched. Higher levels of perceived social support are associated with lower internalizing symptoms (Rueger et al., 2010; Stewart and Suldo, 2011; Väänänen et al., 2014). This has been attributed to high levels of perceived social support providing a protective factor that allows an individual to more effectively handle potentially stressful events (Dumont and Provost, 1999). Perceived social support has been found to be more strongly associated with depressive symptoms compared to anxious symptoms (Haeffel and Mathew, 2010; Rueger et al., 2010; Väänänen et al., 2014). Consistent with these findings, the present results indicate a negative linear association between perceived social support (from family and a significant other) and depressive symptoms; no measure of perceived social support was significantly associated with anxious symptoms.

Perceived social support is multi-factorial (Rueger et al., 2010); therefore we measured perceived social support across three domains relevant to adolescents (friends, family, and a significant other). This allowed us to identify any differences between types of perceived social support and their relationship with motor skills and internalizing symptoms. For the mediation model predicting depressive symptoms, motor skills had an indirect effect on depressive symptoms via perceived family support only. Similarly, perceived family support had the strongest association with depressive symptoms, compared to perceived support from friends and a significant other. The results of the present study indicate that while there is a positive association between motor skills and perceived family support and perceived friend support, only perceived family support was significantly negatively associated with depressive symptoms. The present findings are consistent with previous literature, which has identified family (particularly parents) as the strongest predictor of depressive symptoms and mental health in adolescents (Rueger et al., 2010; Stewart and Suldo, 2011).

This study enlisted a sample of adolescents between 12 and 16 years of age. Consequently, perceived family support may have been the strongest predictor of depressive symptoms due to the central role that family has during early adolescence (Morris et al., 2007). Previous studies have identified a transition in attachment patterns during adolescence, where individuals begin to draw on peers for social support (Noller et al., 2013). The participants in this study were in the early-to-mid stages of adolescent development and the transition from family support to other types of social support may still be underway. It is also equally plausible that perceived family support may be important in an adolescent sample as the family structure (particularly parents) may serve as a “secure base” for individuals to draw support from while they continue to explore and develop peer relationships throughout adolescence (Noller et al., 2013). An awareness of the shifting changes in attachment styles throughout the lifespan provides an important consideration for future studies seeking to explore the relationship between motor skills, perceived social support, and mental health outcomes in different age groups. For example, we may posit that perceived social support from friends, rather than family, may be a stronger predictor of mental health in older adolescent/adult samples. However, other studies have found that perceived family support was a stronger protective factor in depressive symptoms when compared to perceived friend support in adults aged 21–30 years (Pettit et al., 2011). Further empirical investigation is required, specifically within the context of motor skills.

An interesting observation to note was the significant positive association between perceived social support from a significant other and depressive symptoms (see Figure 1), suggesting that higher levels of perceived social support from a significant other is related to higher levels of depressive symptoms, which is inconsistent with previous literature. Additional investigation of this relationship is required in order to determine if these findings can be replicated.

This present study employed a cross-sectional, correlational research design to test a recently proposed causal framework. While the associations between motor skills, perceived family support, and depressive symptoms are congruent with the Elaborated Environmental Stress Hypothesis, the present research design was unable to identify temporal precedence between variables, as data was collected at a single point in time. While changes in perceived social support are generally considered to predispose changes in depressive symptoms, it is important to note that there is some research suggesting that depressive symptoms may influence perceived social support (Leskelä et al., 2008). Consequently, further longitudinal and experimental research is required in order to permit causal conclusions. While we employed robust bootstrapping procedures to address the potential limitation of a sample of 93 participants, enlisting a larger sample of adolescents in future studies is advised, particularly as it will allow for the testing of multiple variables and more rigorous analyses.

## Conclusion

This study evaluated a key pathway specified by the recently proposed Elaborated Environmental Stress Hypothesis, the potential indirect effect of perceived social support between motor skills and internalizing symptoms. We identified an important, direct relationship between motor skills and both depressive and anxious symptoms in a community adolescent sample. There were no indirect effects between motor skills and anxious symptoms via perceived social support from friends, family, or a significant other. However, there was an indirect effect between motor skills and depressive symptoms, via perceived family support only. This study provides partial support for the Elaborated Environmental Stress Hypothesis. The present findings increase our understanding of how motor skills, perceived social support, and internalizing symptoms interact in a community adolescent sample. This has particular implications for the prevention of psychosocial problems in young people with poor motor skills. For example, improving perceived family support may serve as a protective factor that mitigates increased depressive symptoms. Programs aimed at improving the psychosocial outcomes of young people with motor difficulties could potentially include components that seek to improve support from family.

## Author contributions

VM was the primary author of the article, who wrote the majority of the manuscript/conducted analysis. DR was responsible for the larger study of which the current data is part of, and also provided feedback on the manuscript. BH provided supervision and assisted in the methodological procedures of the research. LR and JP provided supervision and feedback regarding the process of the research and the subsequent manuscript.

### Conflict of interest statement

The authors declare that the research was conducted in the absence of any commercial or financial relationships that could be construed as a potential conflict of interest.
